# A Systematic Review and Meta-analysis of the Topical Administration of Fibrin Sealant in Total Hip Arthroplasty

**DOI:** 10.1038/s41598-017-16779-3

**Published:** 2018-01-08

**Authors:** Zhihu Zhao, Xinlong Ma, Jianxiong Ma, Xiaolei Sun, Fengbo Li, Jianwei Lv

**Affiliations:** 1 Orthopaedics Institute, Tianjin Hospital, Tianjin, 300050 People’s Republic of China; 2 Tianjin Hospital, Tianjin University, Tianjin, 300211 People’s Republic of China; 3 Biomechanics Labs of Orthopaedics Institute, Tianjin Hospital, Tianjin, 300050 People’s Republic of China

## Abstract

Patients who undergo total hip arthroplasty (THA) may experience a large amount of blood loss. The objective of our study is to include randomized controlled trials (RCTs) and compare the clinical outcomes of fibrin sealant (FS) versus placebo after a THA. In October 2015, we searched the following databases: Medline, Embase, PubMed, the Cochrane Controlled Trials Register, Web of Science, the China National Knowledge Infrastructure, the China Wanfang database and Google Scholar. Finally, seven studies that included 679 patients met the inclusion criteria. The meta-analysis indicated that the topical administration of FS was associated with a reduction of the need for transfusion compared to the control group (P = 0.05). And topical FS will reduce total blood loss after THA (P = 0.0003) and blood loss in drainage (P = 0.002). However, there was no significant difference in terms of the intraoperative blood loss (P = 0.62) and the rate of deep venous thrombosis (DVT), fever, pain, anemia, hematoma and oozing. In conclusion, the use of FS in patients who are undergoing THA may reduce perioperative blood loss and attenuate the decrease in Hb. Furthermore, FS do not decrease the intraoperative blood loss without an increase in the risk of postoperative DVT, fever, pain, anemia, hematoma and oozing.

## Introduction

Total hip arthroplasty (THA) is one of the most common surgical procedure in orthopedic surgery^1^. However, THA patients may experience a large amount of blood loss ranging from 700 ml to 1700 ml^2,3^. And the up to 37% of THA patients require blood transfusion for postoperative anemia^1^. Blood transfusion was not with zero risk, it has the potential of the transmission of viral diseases and increase cardiac load^4–7^. Many methods (controlled hypotension, hypotensive anaesthesia and administration with desmopressin) have been used to reduce blood loss^8–10^. These methods have drawbacks such as the increase in economic cost and the increase in the chance of thrombosis. Thus, we need to seek an ideal method to reach maximum hemostasis effects without increasing economic costs and complications.

Fibrin sealant (FS) is manufactured from human plasma products and composed of human fibrinogen and thrombin^11,12^. Studies that have examined the effect of FS on blood loss during THA reveal conflicting results: two randomized controlled trials (RCTs) reported that FS significantly reduced postoperative blood loss without affecting the need for transfusion^13,14^, while another RCT^15^ found no effect on either parameter and an early retrospective study reported a reduced need for transfusion only^16^. Therefore, a meta-analysis was conducted to provide evidence from RCTs to evaluate the efficiency and safety of topical FS in patients who were undergoing primary THA. In addition, this paper hypothesizes topical use of FS has a positive role on reducing blood loss without increasing the occurrence of deep venous thrombosis (DVT), fever, pain, anemia, hematoma and oozing.

## Results

### Search results

**Figure 1:**
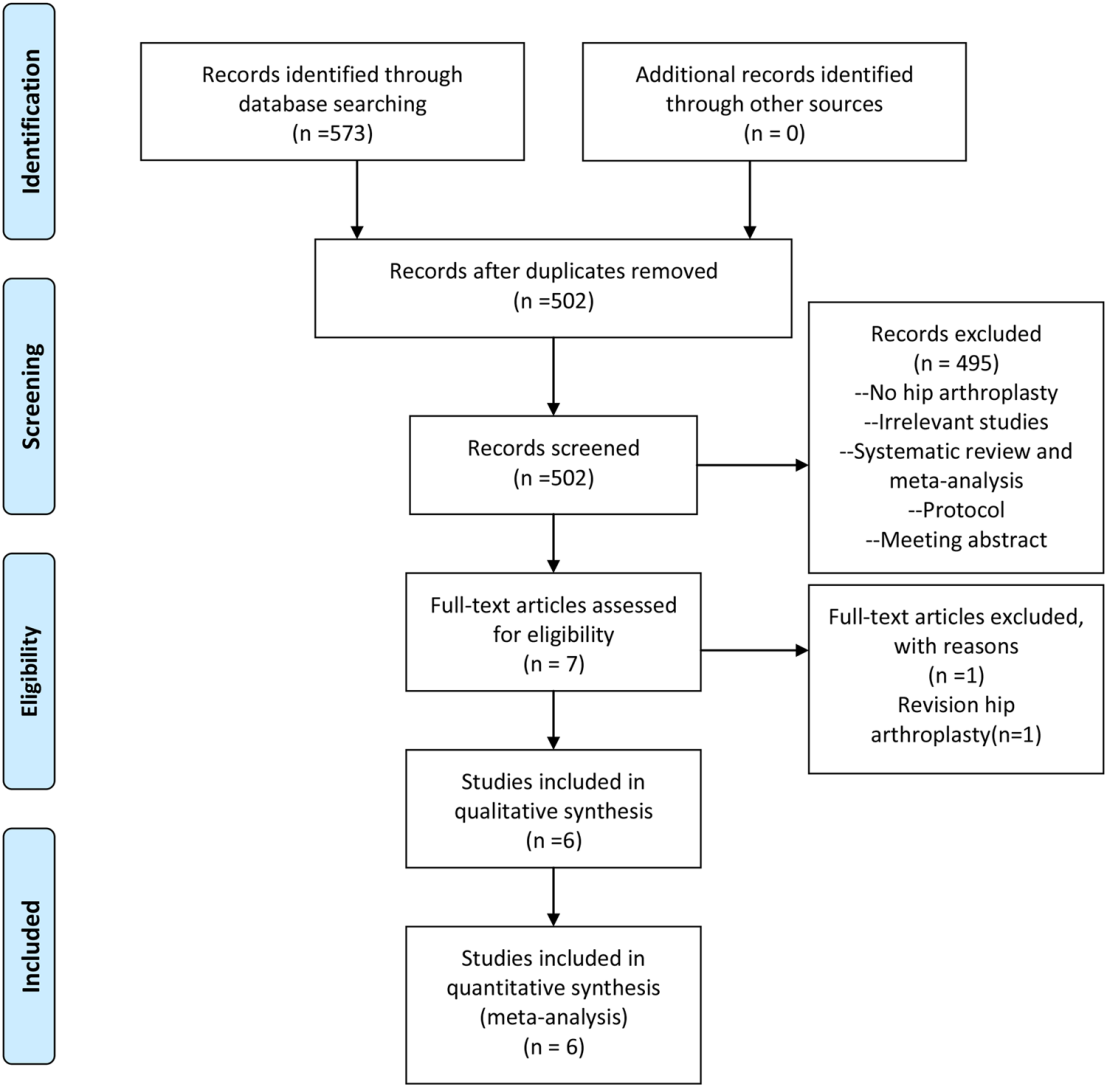
Flow diagram of study selection.

**Figure 2:**
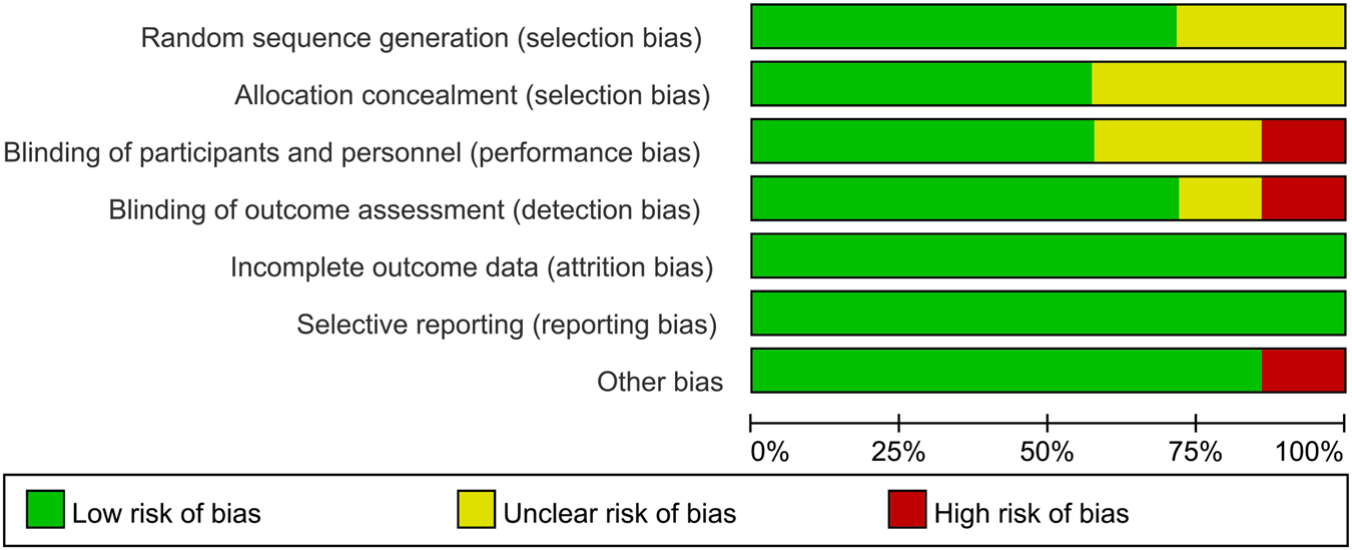
The risk of bias graph.

**Figure 3:**
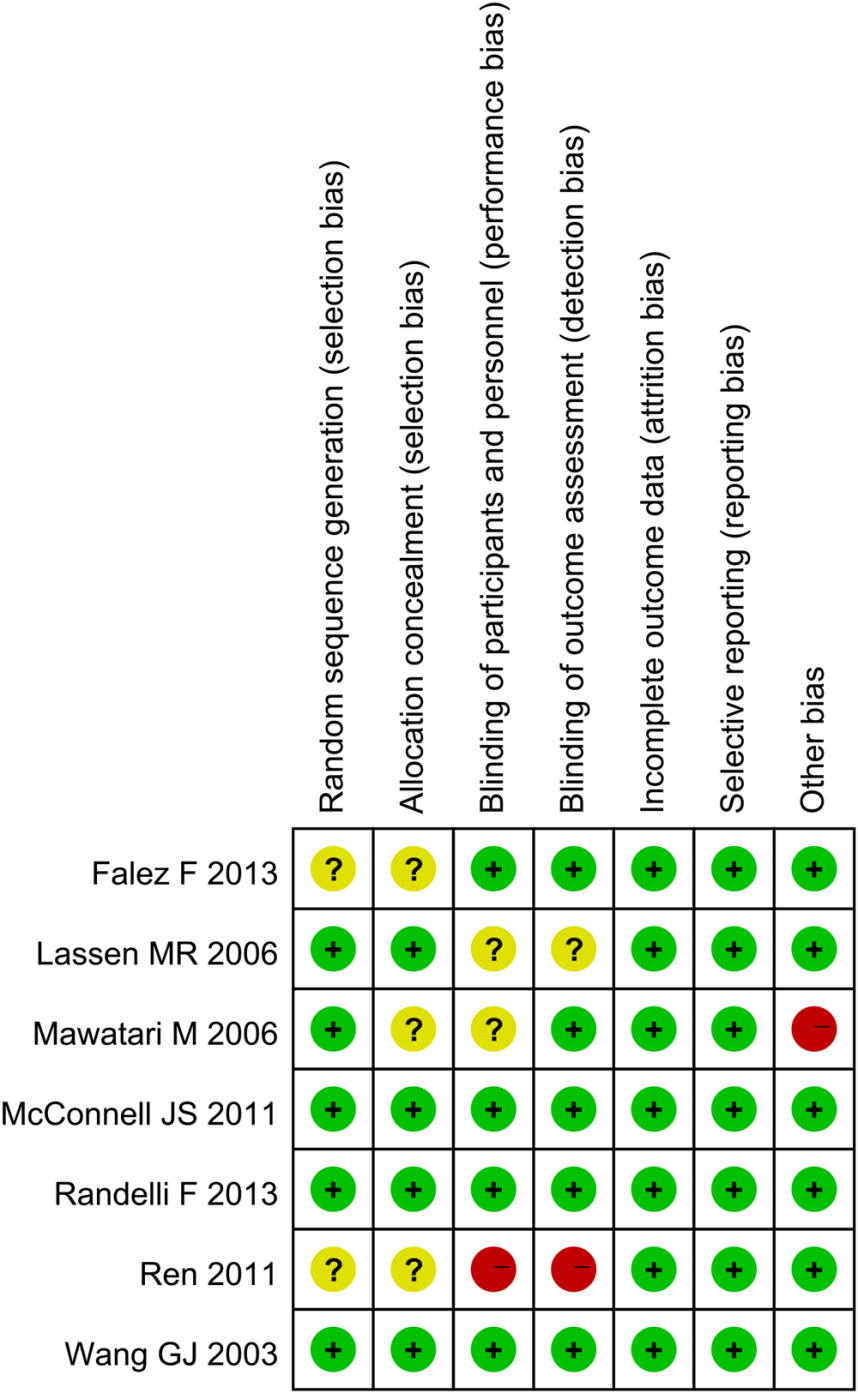
The risk of bias of summary.

Figure 1 presents a summary of the study selection process. Firstly, we identified 573 potentially relevant studies according to the search strategies. According to the inclusion and exclusion criteria, we finally included seven RCTs with 679 patients (679 hips) in the meta-analysis^13–15,17–20^. Of the included studies, six articles were in English and one was in Chinese. All of the included RCTs were published from the year of 2003. The characteristics of the seven RCTs were presented in Table 1. The mean age of the THA patients in the included RCTs ranged from 60.0 to 75.1 years. The dose of FS was 5 ml to 10 ml. The origin of FS was from four products: auto blood, Quixil, Yueling Jiao and Omrixil. In addition to one trial that used the uncemented prostheses, the remainder of the studies all included cemented prostheses. All of the studies detailed the operative approach and the use of tourniquets. Three trials referenced the transfusion criteria. Quality assessments of the studies are shown in Figs 2 and 3.

**Table 1:** The general characteristic of the included studies. FS, fibrin sealant; Co, control; Yueling Jiao, a commercial fibrin sealant made in China that is derived from pigs; NS, not stated; Y, yes; N, no.

Study (Year)	Cases (FS/Co)	Mean Age (FS/Co)	Male/Female (FS/Con)	Doses	Origin of FS	Operation Methods	Drainage	DVT Prophylaxis	Type of Prosthesis
Lassen^15^	33/36	67.1/63.1	12/21, 15/21	NS	Autologous blood	NS	Y	NS	Cemented/ Cementless
Falez^17^	31/38	NS	NS	10 mL	Quixil	lateral	NS	Enoxaparin 4000 U	Cementless
Wang^13^	38/43	66.9/67.8	22/16, 23/20	10 mL	Omrixil	anterior or lateral	Y	Warfarin 10 mg/day	Cemented/Cementless
McConnell^18^	22/22	NS	5/17, 9/13	10 mL	Quixil	NS	N	Aspirin 150 mg	Cemented
Mawatari^14^	50/50	60/60	100/0	10 mL	Autologous blood	lateral	Y	NO	Cementless
Randelli^19^	21/21	NS	NS	10 mL	Quixil	lateral	Y	Enoxaparin NS	Cementless
Ren 2011	20/20	75.1	14/26	5 mL	Yueling Jiao	lateral	Y	NS	NS

### Results of the meta-analysis

#### Need for transfusion

**Figure 4:**
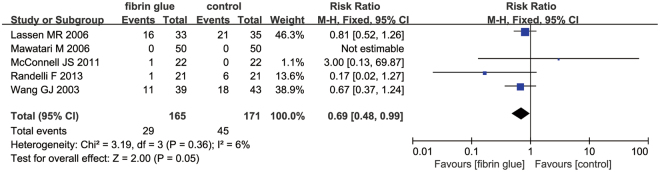
The forest plot of need for transfusion between the two groups.

Only five studies with 336 patients provided data about the need for transfusion between FS group and control group. The meta-analysis revealed that the topical FS was associated with a reduction of the need for transfusion compared to the control group (Fig. 4) (RR = 0.69; 95% CI 0.48 to 0.99; P = 0.05).

#### Total blood loss, blood loss in drainage and intraoperative blood loss

**Figure 5:**
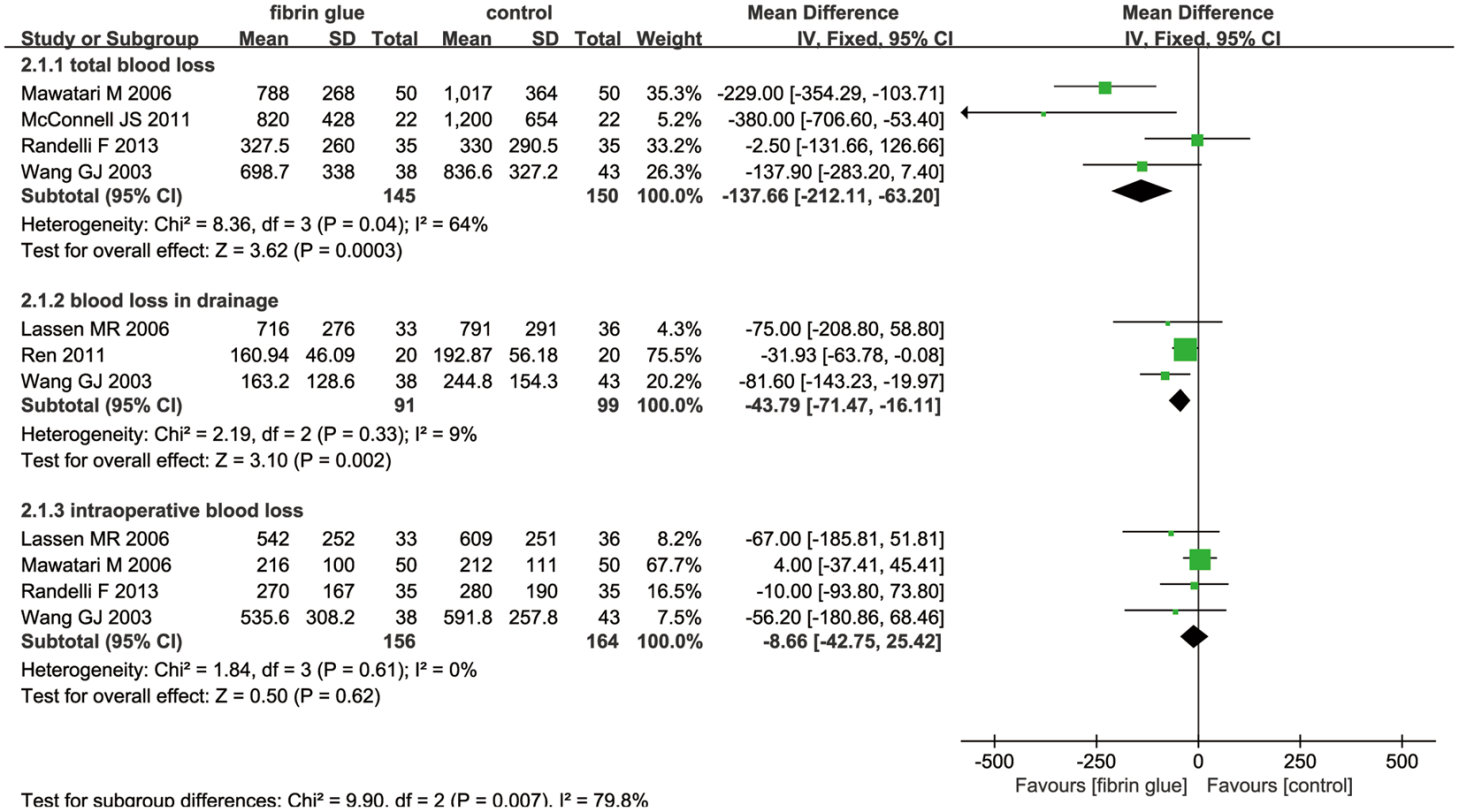
The forest plot of total blood loss, blood loss in drainage and intraoperative blood loss between the two groups.

Only four studies with 295 patients reported the total blood loss after the application of FS. The meta-analysis revealed that the topical application of FS reduced the total blood loss after THA (Fig. 5) (MD = −137.66; 95% CI −212.11 to −63.20; P = 0.0003).

A total of three component studies (190 patients) reported blood loss in drainage after THA. Topical FS was associated with a reduction of blood loss in drainage by 43.79 ml (Fig. 5) (MD = −43.79; 95% CI −71.47 to −16.11; P = 0.002).

Four studies reported the intraoperative blood loss after THA. Meta-analysis result indicated that use of FS was not associated with a reduction of the intraoperative blood loss (MD = −8.66; 95% CI −42.75 to 25.42; P = 0.62) (Fig. 5).

#### The decrease in Hb

**Figure 6:**
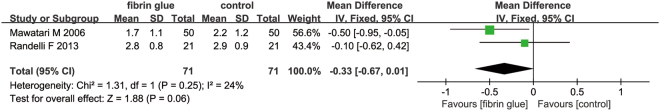
The forest plot of the decrease in Hb levels between the two groups.

Two RCTs with 142 patients were included on the decrease in Hb. Results indicated that topical FS was not associated with a reduction of the decrease in Hb (Fig. 6) (MD = −0.33; 95% CI −0.67 to 0.01; P = 0.06).

### Complications

**Figure 7:**
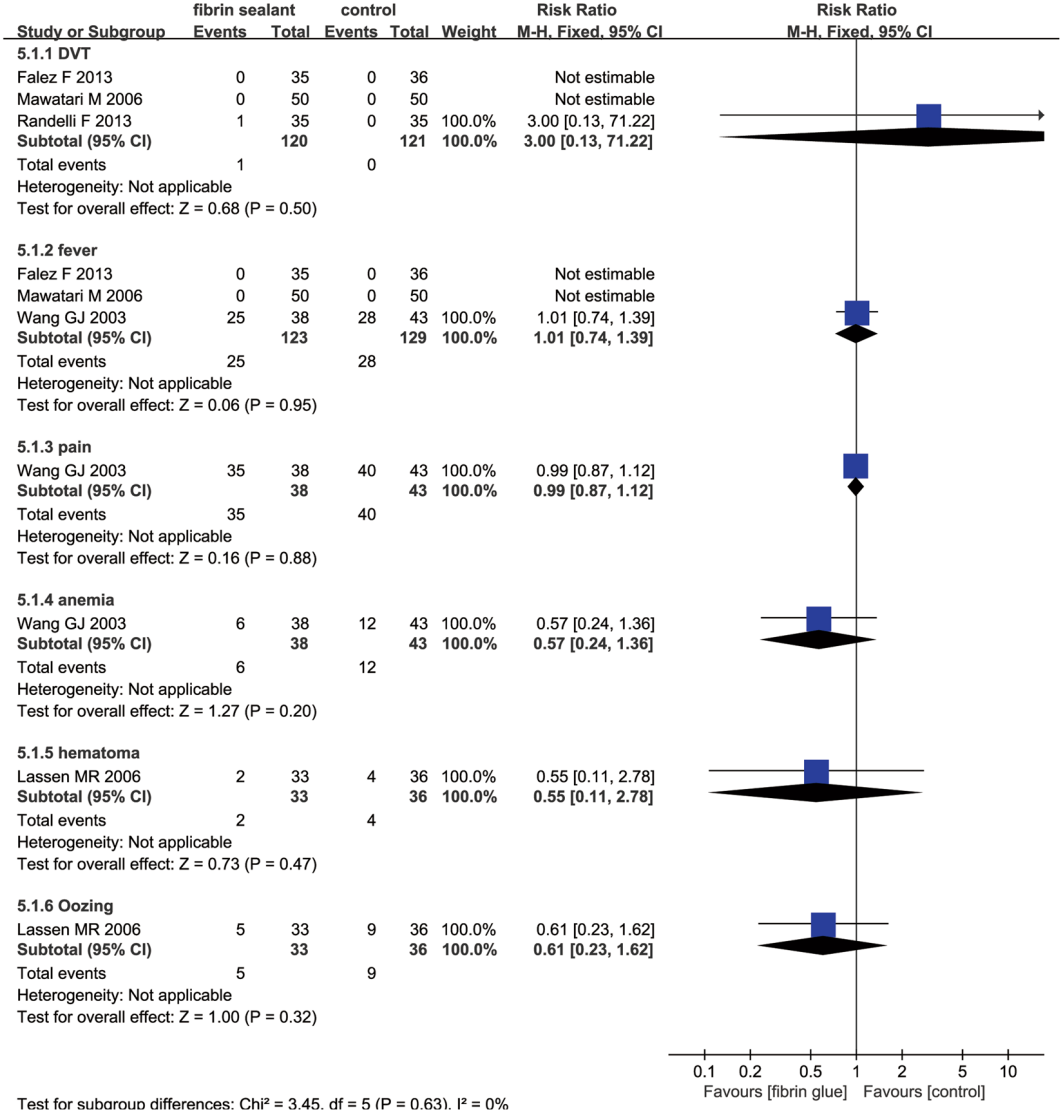
The forest plot diagram showing the effect of a FS on adverse events.

Three studies paid close attention to the occurrence of deep venous thrombosis (DVT). The meta-analysis reported no significant difference in the occurrence of DVT, fever, pain, anemia, hematoma and oozing (Fig. 7).

## Discussion

This is the first meta-analysis that only included RCTs and compared topical use of FS in the controlling intra-operative and post-operative blood loss after THA. Results indicated that FS can reached better blood loss control than controls after THA. As for the need for transfusion, the occurrence of DVT and other complications, there was no significant change between the FS group and controls. One study was from 2003, and the remainder of the studies were from 2006. All of the included RCTs were with high quality; only two studies did not use randomized control trial methods^17,20^, and three studies did not report their concealment method^14,17,20^. The blinding of participants and personnel was unclear in two studies^14,15^ and was a high risk in one study^20^. The blinding of outcome assessment was unclear in one study^15^ and was a high risk in one study^20^. The double-blind method was used in all of the RCTs and all of the included studies showed comparable baseline data and provided the intention to treat analysis.

Better blood loss management is especially important for THA patients, as blood loss may lead to tachycardia and can increase the risk of mortality. Blood transfusion may be cost-effective but may increase the risk of infection^21–23^. FS, an effective hemostasis agent, has been used to reduce peri-operative blood loss in various surgical methods^24^. However, the efficacy of FS in decreasing the blood loss after THA is still controversial. As a result, a systematic review and meta-analysis are essential in the identification of the effect of topical FS on blood loss management after THA.

Final results indicated that topical FS can decrease the need for transfusion by 8.74%. The rate of the need for transfusion in the FS group was 17.58% (29/165) and in the control group was 26.32% (45/171). The number of people who were engaged in the research was not large enough and the differences may have reached significance in a larger clinical trial. The results revealed that the topical spray FS can decrease the total blood loss by 137.66 ml and the blood loss in drainage by 43.79 ml; however, there was no significant difference in the intraoperative blood loss between the two groups (MD = −8.66; 95% CI −42.75 to 25.42; P = 0.62). Only one study calculated blood loss and applied a formula described by Jeffrey B. Gross^17^. The remaining studies calculated blood loss from suction drains and swabs, and this may underestimate the real degree of peri-operative bleeding. Otherwise, all of the trials did not collect the hidden blood loss, which may have contributed to a large proportion of the total blood loss^25^. Many meta-analyses have published and identified that the topical FS has a positive role in decreasing blood loss in total knee arthroplasty (TKA)^26,27^. However, due to the large area of bone cutting and drainage, the blood loss in THA is larger than in TKA. The origin of FS is mainly from four products: autologous blood, Quixil, Omrixil and Yueling Jiao; of 7 studies, two trials used autologous blood to obtain FS^14,15^, three studies used commercial Quixil^17–19^, one study used Omrixil^13^, and one study used Yueling Jiao^20^. Ren *et al*. used 5 ml of FS to reach hemostasis outcome and one study did not state the volume of FS; the remainder of the studies used 10 ml of FS to decrease blood loss.

Since Falze *et al*.^17^ only reported the mean value of blood loss without providing the standard deviation, we tried to contact the author to obtain the standard deviation; however, we received no reply. These data could not be included in the final results and will affect the final results. Randelli *et al*.^19^ used tranexamic acid during the immediate preoperative period; since tranexamic acid is also a hemostasis agent, this will also affect the final conclusion.

As for complications, there are primarily six complications that are associated with the administration of FS, including DVT, fever, pain, hematoma, anemia and oozing. For clinical physicians, DVT may be considered to be one of the most dangerous complications and secondary pulmonary embolism may be a deadly complication. Our results revealed that there was no significant change between the FS group and control group as regard to the occurrence of DVT. This finding may be because FS is sprayed locally and thus does not influence systemic coagulation. Theoretically, the use of FS during THA reduces the postoperative blood loss that permeates into the tissues to form hematomas; however, there was no significant difference in the occurrence of hematoma between the FS group and control group. This finding indicates that FS may have no benefit for hidden blood loss. Other identified trials were insufficient for this meta-analysis; Mawatari *et al*.^14^ revealed that FS has no effects on the rate of bone ingrowth and heterotopic ossification when compared control group at 3-year follow-up.

The main problems with FS are the cost and the biological risks (human-derived products and animal-derived products). In recent years, however, cheaper hemostasis strategies (such as tranexamic acid, TXA) have appeared, and may surpass fibrin spray in terms of costs and biological risks^28^. McConnell *et al*.^29^ reported that the FS was cheaper than TXA (£3.10 for TXA and £390 for FS) in TKA. Similarly, McConnell *et al*.^18^ found that TXA can achieve equivalent hemostasis effects to FS without increasing the hospitalization costs, TXA may be superior to FS. This superiority may demand the performance of high quality RCTs for verification.

The pooled data of our meta-analysis have several limitations: (1) 7 RCTs with 679 patients were finally included in this meta-analysis, so the sample size is too small to get an accurate result; (2) the heterogeneity of some results (total blood loss and the decrease in Hb) were relatively high, and this will affect the precision of the results; (3) we did not performed publication bias due to the limited number of the included studies (less than 10 studies), and publication bias may existed in this meta-analysis; and (4) the dose of FS application were different from each other, which need for more studies to identify the optimal therapeutic dose of FS.

## Conclusion

Our meta-analysis results revealed that the topical application of FS can decrease intra-operative and post-operative blood loss without an increase in the complications compared to the placebo. The most important finding of this study is that FS did not decrease the need for transfusion and did not affect the decrease in Hb levels. For future research, optimal drugs and drug dosages should be rigorously defined, and the method of spray FS should also be clarified.

## Methods

This review is registered in Protocol registration: PROSPERO 2016:CRD42016035748.

### Search Strategy

Electronic databases, including Medline, Embase, PubMed, the Cochrane Controlled Trials Register, Web of Science, the China National Knowledge Infrastructure, the China Wanfang database and Google Scholar were searched in October 2015 to identify relevant studies that compared the topical use of FS for blood loss controlling during THA. The search keywords and corresponding medical subject heading (Mesh) terms were “fibrin sealant,” “fibrin adhesive tissue,” “fibrin glue,” “total hip arthroplasty,” “total hip replacement” “THA” “THR”. The details of the search strategies are presented in Supplementary Table S1. Additionally, we read the reference for potential eligible reports. There were no restrictions in terms of the date or language of publication.

### Eligibility criteria

Inclusive criteria was as follows: (P) patients who were undergoing primary THA; (I) studies that used topical FS as a therapy to control bleeding; (C) studies that used saline or nothing as control group; (O) outcomes such as blood loss in drainage, total blood loss, the need for transfusion, the rate of deep venous thrombosis (DVT) and infection; and (S) studies that were designed as randomized controlled trials (RCTs).

### Study quality assessment

According to the Cochrane Collaboration tool^30^, we performed the risk of bias for each RCTs. A total of seven items were used for assessing the risk of bias. Two reviewers (Z.Z. and X. M.) were trained and independently assessed the quality of the included RCTs and any divergence were settled by discussion.

### Data extraction

Once the duplicates were excluded, two reviewers (J.M. and F.B.) independently extract data from the eligible RCTs. Extracted data (general characteristic of the patients, the dose and origin of FS, operation methods, the adoption of drainage and DVT prophylaxis and type of prosthesis; and outcomes) were entered into a pre-generated standard Microsoft^®^ Excel. If the data were presented as figure or other form, we extracted them with the Software “Getdata Graph Digitizer”.

### Statistical analysis

The primary outcomes were the need for transfusion, total blood loss, blood loss in drainage, the decrease in Hb, and the rate of DVT. The second outcome measures were the intraoperative blood loss, the occurrence of fever, pain, anemia, hematoma and oozing. Mean differences (MD) with their respective 95% confidence intervals (CIs) for FS compared with controls were calculated for continuous outcomes (blood loss in drainage, intra-operative blood loss, total blood loss and the decrease in hemoglobin). Relative risk (RR) with 95% CIs for FS compared with controls were calculated for discontinuous outcomes (the occurrence of fever, pain, anemia, hematoma, oozing, DVT and the need for transfusion). RevMan 5.3 Software (The Nordic Cochrane Centre, The Cochrane Collaboration, Copenhagen, 2014) was used for calculating meta-analysis. Chi-squared test results and I^2^ statistic were used for measuring the heterogeneity. P > 0.1 or I^2^ ≥ 50% were considered statistical heterogeneity. A meta-analysis was performed with fixed effect (P < 0.1, I^2^ < 50%) or random effect models (P > 0.1, I^2^ ≥ 50%). P < 0.05 was identified as statistical significance.

## Electronic supplementary material

Supplement S1
