# Supplementary material for: A Systematic Review and Meta-analysis of the Topical Administration of Fibrin Sealant in Total Hip Arthroplasty
Source: Sci Rep. 2018 Jan 8;8:78. doi: 10.1038/s41598-017-16779-3 (PMC5758515; doi:10.1038/s41598-017-16779-3)
Supplement: Supplementary file 1 — Supplement S1 [file 41598_2017_16779_MOESM1_ESM.pdf]

# A Systematic Review and Meta-analysis of the Topical Administration of Fibrin Sealant in Total Hip Arthroplasty

Zhihu Zhao[a,b], Xinlong Ma[a,b], Jianxiong Ma[c], Xiaolei Sun[a,b], Fengbo Li[a,b], Jianwei Lv[a,b]

a Orthopaedics Institute, Tianjin Hospital, Tianjin, 300050, People's Republic of China.

b Tianjin Hospital, Tianjin University, Tianjin, 300211, People's Republic of China.

c Biomechanics Labs of Orthopaedics Institute, Tianjin Hospital, Tianjin, 300050, People's Republic of China.

Corresponding author. Xinlong Ma, Address: Orthopaedics Institute, Tianjin Hospital, NO.406 Jiefangnan Road, Tianjin, 300000, China. Fax: + 86 13516118843. E-mail: tianjinbone@163.com.

| **Search** | **Query** |
|---|---|
| #1 | "Fibrin Tissue Adhesive"[Mesh] |
| #2 | fibrin glue |
| #3 | fibrin sealant |
| #4 | fibrin adhesive tissue |
| #5 | ((arthroplast*[Title/Abstract]) OR replac*[Title/Abstract]) OR prosthe*[Title/Abstract] |
| #6 | "Hip Joint"[Mesh] OR "Hip"[Mesh] |
| #7 | (hip[Title/Abstract]) OR hip joint[Title/Abstract] |
| #8 | "Arthroplasty, Replacement, Hip"[Mesh] |
| #9 | "Hip"[Mesh] OR "Hip Joint"[Mesh] |
| #10 | (hip[Title/Abstract]) OR hip joint[Title/Abstract] |
| #11 | #1 OR #2 OR #3 OR #4 |
| #12 | #5 OR #6 OR #7 OR #8 OR #9 OR #10 |
| #13 | "Randomized Controlled Trial" [Publication Type] OR "Randomized Controlled Trials as Topic"[Mesh] |
| #14 | random* |
| #15 | #13 OR #14 |
| #16 | #11AND #12 AND #13 |

**PubMed**

**Searched on:** August 2015

**Results:** 78

**Supplementary Table S1**: Search strategies.
